# Evolution, Bioinformatics and Evolutionary Bioinformatics Online

**Published:** 2007-01-15

**Authors:** Mark Pagel

**Affiliations:** Editor-in-Chief, Evolutionary Bioinformatics Online Reading, United Kingdom

E*volutionary Bioinformatics Online* enjoyed a busy and productive first year in 2005, capped off by being accepted by the Literature Selection and Technical Review Committee at the National Library of Medicine in the US, for inclusion in PubMed Central. This means that EBO will be indexed online in this internationally preeminent archive.

A brief history. *Evolutionary Bioinformatics Online* was established as the official journal of The Bioinformatics Institute, a joint-venture between the University of Auckland, situated in New Zealand’s largest city, and AgResearch, New Zealand’s largest Crown Research Institute. Allen Rodrigo, Professor of Computational Biology and Bioinformatics, is the Institute’s Director, and it was at his initiative that the journal was established. Rodrigo, The Institute, and I all work with Libertas Academica, a publishing firm committed to high editorial standards and open access publishing methodologies, to produce the journal. We are fortunate for the help of an impressive editorial board (http://www.la-press.com/EBO-edboard.htm), and our acceptance into PubMed Central in our first year reflects the board’s reputation and high standard of submissions accepted for publication.

The contents of our Volume 1 gives some views of where our field is now: papers on phylogenetics, genetic databases and software for managing and exploiting them, gene regulation, and proteomics. These papers show how evolutionary biologists are working with researchers in these areas to begin to produce working pictures of how genomes and gene-regulation evolve. This situation will only grow more complex as more and more species are characterised at the whole-genome level. New architectures and technologies for building very large trees — grid computing, supertrees, and parallel algorithms for tree building – will be needed, as will software for conducting comparisons among them.

Looking ahead what topics might come to dominate future volumes of the journal? My sense is that from the beginning just described, a big challenge for evolutionary bioinformatics is to begin to unravel the evolution of phenotypes. This means understanding networks of genes, their topological properties, how the networks evolve, how their genes are regulated and, finally, how these wired-up and regulated networks produce the phenotypes we see. This is the real *evo-devo* (evolutionary developmental biology) we have been waiting for. It will depend on good phylogenies, good models of sequence and protein evolution and on the efforts of the armies of people annotating genes, and studying their expression with such technologies as microarrays or *RNAi.*

The attractiveness of this view for evolutionary biologists is that it is so well suited to the many other things we do. Evolutionary biologists have spent almost the last 150 years studying history. It may be time to shift to “rules” and “laws” of evolution – what can we predict will evolve, or at least what can we predict about how things will evolve? Given some understanding of how phenotypes map onto genotypes, and how genotypes evolve will underpin progress in this area, and may give insight into why some attempts at prediction based solely on sequence – such as those for the influenza virus – have had limited success. Astrobiologists might wish to pay attention: how organisms work on this planet and why, should give some insights into how they might be put together in different environments. This knowledge could prove useful in the design of sensors to detect life.

New sequencing technologies are going to see the field of population genomics emerge – large samples of genomes from individuals in a population. Microbiologists, note that you may finally be able to test just how promiscuous microbes are! Going a step deeper, environmental sequencing promises (threatens?!) to identify hundreds of thousands or perhaps millions of new genes: Buzz Lightyear’s aphorism “to infinity and beyond” may be more true than we care to imagine just now. It is going to take creative thinking and methods to help to assemble these environmental samples into putative genomes and species.

I say ‘threaten’ above because already there is something of a ‘functional genomics gap’ between the genes that have been identified and our knowledge of what they do. Coalescent modeling combined with the analysis of selection among populations can bring the understanding of phenotypes right down to the level of variation within populations. It can also identify important genes associated with differential survival in varying habitats, conferring disease resistance, or associated with recovery from environmental shock. This kind of population level thinking combined with phenotypes may also help to identify principles of cross-species transmission and the spread of infectious agents within and between populations. For example, there has been some progress in identifying how the protein network of an infectious agent merges topologically with its host’s protein network.

The promising conclusion from this is that there is far more for evolutionary biologists and bioinformatics researchers to do than they possibly can. Equally encouraging is that the issues this work confronts are some of the most pressing we have: aging populations will mean that understanding phenotypes and how to alter them could be valuable in improving quality of life and saving money for health care systems; understanding how populations evolve and adapt, and linking phenotypes to environmental change may prove useful as we seemingly sleep-walk into global warming; and as the current vigilance about ‘bird flu’ reminds us, even in our high-tech and sanitized modern era, infectious diseases still kill more people on the planet every year than any other cause. Elsewhere (Pagel, 2002) I have suggested that biology is the physics of the 21^st^ century, capturing the public’s attention and garnering a large share of government and private funding for science. These major issues confronting humanity, all with a biological content, show why.
